# The Role of the Oxidative State and Innate Immunity Mediated by TLR7 and TLR9 in Lupus Nephritis

**DOI:** 10.3390/ijms242015234

**Published:** 2023-10-16

**Authors:** Raquel Echavarria, Ernesto Germán Cardona-Muñoz, Pablo Ortiz-Lazareno, Jorge Andrade-Sierra, Luis Francisco Gómez-Hermosillo, Jorge Casillas-Moreno, Tannia Isabel Campos-Bayardo, Daniel Román-Rojas, Andrés García-Sánchez, Alejandra Guillermina Miranda-Díaz

**Affiliations:** 1 Centro de Investigación Biomédica de Occidente, Instituto Mexicano del Seguro Social, Guadalajara 44340, Mexico; raquel.echavarria.z@gmail.com (R.E.); pablolazareno@gmail.com (P.O.-L.); 2 Investigadores por México, Consejo Nacional de Ciencia y Tecnología (CONACYT), Ciudad de México 03940, Mexico; 3 Department of Physiology, University Center of Health Sciences, University of Guadalajara, Guadalajara 44360, Mexico; cameg1@gmail.com (E.G.C.-M.); jorg_andrade@hotmail.com (J.A.-S.); luisgomez_53@hotmail.com (L.F.G.-H.); jcasillas_moreno@hotmail.com (J.C.-M.); tanniaisabelcb@gmail.com (T.I.C.-B.); daniel.rrojas@academicos.udg.mx (D.R.-R.); andres_garciasanchez_3@hotmail.com (A.G.-S.)

**Keywords:** systemic lupus erythematosus, lupus nephritis, antioxidants, oxidative stress, DNA damage, mitochondrial function, mitophagy

## Abstract

Lupus nephritis (LN) is a severe complication of systemic lupus erythematosus (SLE) and is considered one of the leading causes of mortality. Multiple immunological pathways are involved in the pathogenesis of SLE, which makes it imperative to deepen our knowledge about this disease’s immune-pathological complexity and explore new therapeutic targets. Since an altered redox state contributes to immune system dysregulation, this document briefly addresses the roles of oxidative stress (OS), oxidative DNA damage, antioxidant enzymes, mitochondrial function, and mitophagy in SLE and LN. Although adaptive immunity’s participation in the development of autoimmunity is undeniable, increasing data emphasize the importance of innate immunity elements, particularly the Toll-like receptors (TLRs) that recognize nucleic acid ligands, in inflammatory and autoimmune diseases. Here, we discuss the intriguing roles of TLR7 and TLR9 in developing SLE and LN. Also included are the essential characteristics of conventional treatments and some other novel and little-explored alternatives that offer options to improve renal function in LN.

## 1. Introduction

Systemic lupus erythematosus (SLE) is a chronic autoimmune disease mainly affecting women of childbearing age. SLE has a broad spectrum of clinical manifestations involving multiple organs and systems [1]. Over 90% of SLE patients experience inflammatory joint pain with swelling at some point during their disease [2]. Meanwhile, 60–90% of patients develop inflammatory skin rashes, and in 10–30% of patients, inflammation of the serous membranes results in pleuritis or pericarditis [3]. Hematological abnormalities, including leucopenia, thrombocytopenia, and autoimmune hemolytic anemia, are also observed in patients with SLE [4]. Estimates suggest that the kidneys are affected in 35–60% of SLE patients, whereas neuropsychiatric manifestations occur in 39–50% [5,6].

SLE patients typically experience periods of high activity, flares, and remission. Fever, fatigue, arthralgia, and weight loss are nonspecific symptoms of new or recurrent active SLE. Regardless of treatment, flares and disease activity occur chronically in most SLE patients, and cardiovascular disease (CVD) is a common cause of morbidity and mortality [7]. Atherosclerosis and antiphospholipid antibodies (aPL) leading to antiphospholipid antibody syndrome (APS) are two underlying mechanisms of CVD in these patients [8,9]. Oxidized low-density lipoprotein and a pro-inflammatory T-cell profile are shared characteristics of atherosclerosis and SLE. In contrast, aPL can interfere with coagulation, often leading to arterial and venous thrombosis.

**Figure 1:**
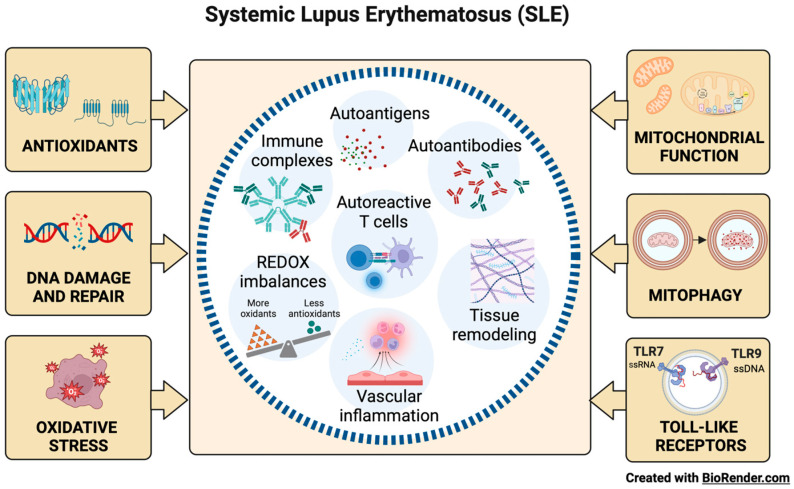
Factors in SLE development. Microvascular inflammation with the development of autoantibodies, pathogenic immune complexes, and autoreactive T cell activation against ubiquitous nuclear antigens are characteristic of SLE. Oxidative stress (OS), antioxidant capacity, DNA damage and repair, mitochondrial function, mitophagy, and innate immunity mechanisms mediated by the toll-like receptors TLR7 and TLR9 influence the induction of systemic autoimmunity and tissue damage.

Microvascular inflammation and autoantibody development against nuclear components are characteristic of SLE (Figure 1) [7]. Autoantibodies and autoreactive T cell presentation occur against ubiquitous nuclear antigens like chromatin and ribonucleoproteins, unleashing systemic autoimmunity, inflammation, and tissue remodeling. Altering endogenous antigens, coupled with autoantibody production, promotes the formation of pathogenic immune complexes and organ damage [10].

## 2. Oxidative Stress in SLE

Oxidative stress (OS) results from an imbalance between the synthesis and neutralization of reactive oxygen species (ROS) and correlates with autoimmune disease activity [11,12]. The interaction of ROS with carbohydrates, lipids, proteins, and nucleic acids triggers autoimmunity and tissue damage through abnormal exposure to endogenous antigens, immunomodulation, and cell death signal responses (Figure 1) [13,14]. 

Endogenous ROS, formed by mitochondria and extramitochondrial organelles such as the endoplasmic reticulum (ER), and exogenous ROS from UV radiation, chemical exposure, and viral or bacterial infections are involved in SLE onset [15]. The electron transport chain generates ROS as part of the mitochondria’s oxidative phosphorylation (OxPhos) process. Meanwhile, nicotinamide adenine dinucleotide phosphate (NADPH) oxidase (NOX), nitric oxide synthase (NOS), and xanthine oxidase (XO) are the leading causes of extra mitochondrial OS [16]. The superoxide anion radical (O_2_•−) and hydrogen peroxide (H_2_O_2_) are among the main ROS regularly accompanied by proinflammatory signals and can interact with nitric oxide (NO) to generate reactive nitrogen species (NRS), like the highly reactive nitrosonium cation (NO+), nitroxyl anion (NO−) and peroxynitrites (ONOO−) [17].

Lipids in mitochondria, lysosomes, and cell membranes are susceptible to oxidation due to the double bonds of unsaturated fatty acids and serve as the major sites of OS, allowing for increased lipid peroxidation (LPO) in disease [18]. The LPO cascade generates many breakdown end-products, including reactive aldehydes such as malondialdehyde (MDA), MDA-modified proteins, 4-hydroxy-nonenal (HNE), HNE-modified proteins, and levels of F2-isoprostanes [19]. LPO generates a wide variety of metabolites, the best known being the saturated mono aldehydes, unsaturated aldehydes, dicarbonyls, MDA, 4-oxo-2-nonenal, hydroxydialdehydes (HNE, 4-hydroxy-2-hexenal), and oxidized phospholipids [20]. Humans limit the destructive effect of LPO through LPO metabolization by oxide reductase enzymes (aldo-keto-reductase, aldehyde-dehydrogenase, alcohol-dehydrogenase, Glutathione-S-Transferase) and cellular antioxidant defense mechanisms that include enzymes such as superoxide dismutase (SOD), catalase (CAT), glutathione peroxidase (GPx), glutathione reductase (GR), thioredoxin reductase, heme-oxygenase, and vitamins A, C and E, carotenoids, flavonoids, glutathione (GSH) and other antioxidant minerals [21].

The free radical-mediated peroxidation of arachidonic acid generates isoprostanes independent of cyclooxygenase activity; thus, plasma F2-isoprostanes measure the balance between ROS production and antioxidant defenses [22]. Currently, the measurement of F2-isoprostanes in plasma is considered the most reliable index of OS in vivo due to their specific structural characteristics and relative chemical stability, and high levels of F2-isoprostanes are associated with oxidative tissue damage in various autoimmune disorders [23,24]. In SLE, plasmatic levels of F2-isoprostane correlate with high disease activity, renal manifestations, mitochondrial function, and APS anti-β2-glycoprotein I (anti-β2GPI) presenting with multiple clinical phenotypes and complications [25,26].

**Figure 2:**
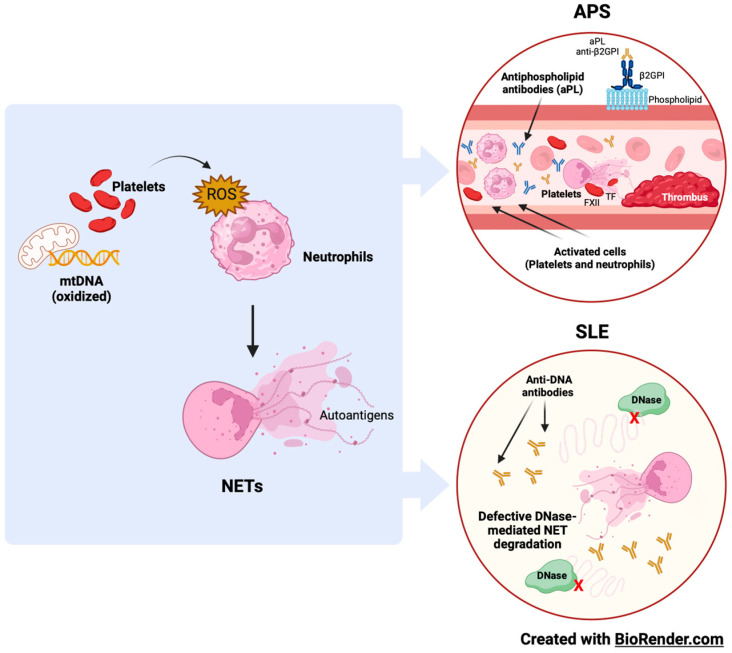
Oxidative stress, neutrophils, and NETs in APS and SLE. Neutrophils’ immune functions, including NETosis, are primarily mediated by ROS. Neutrophil–platelet interactions augment ROS production and ease NET release. Autoantibodies against phospholipids and DNA are typical of APS and SLE, and components released by NETs often contribute to the antigenic load.

SLE and APS neutrophils present an activated phenotype [27]. Neutrophil activation induces excess ROS production, essential for forming neutrophil extracellular traps (NETs) through the induction of extensive DNA damage and repair that leads to chromatin decondensation [28]. The immune system of vulnerable individuals can recognize components released by NETs as autoantigens, thus influencing the pathogenesis of inflammatory and autoimmune diseases. Patients with SLE, but not APS, have defective DNase-mediated NET degradation [29]. In contrast, anti-β2GPI autoantibodies induce NETs and enhance thrombosis through their ability to bind and activate platelets, tissue factors, and coagulation factor VII [30] (Figure 2).

Furthermore, data from a multinational cohort of aPL antibody-positive patients without lupus evidenced high levels of anti-NET antibodies in 45% of aPL-positive patients, likely targeting NET-associated protein antigens [31].

## 3. DNA Damage and Repair in SLE

The most common form of oxidative DNA damage is the formation of 8-hydroxy-2′-deoxyguanosine (8-OHdG), which, without repair, can result in adenine (A) mispairing with 8-OHdG instead of cytosine (C) and cause guanine (G): C to T: A cross-mutation in human cells [32]. Hence, 8-OHdG is a DNA marker that reflects the oxidative status of the whole organism, and that accumulates in tissues and induces genomic instability and cellular dysfunction [33]. Moreover, 8-OHdG is a sensitive and specific predictor of oxidative DNA damage in SLE flares (Figure 1). 8-OHdG can be repaired primarily by the human enzyme 8-oxo guanine DNA glycosylase 1 (hOGG1) through base excision repair mechanisms. hOGG1 performs preventive actions against inflammatory responses by reconfiguring 8-OHdG levels [34,35]. 

## 4. Antioxidants in SLE

Free radicals (FR) and other ROS can interact with all the biomolecules present in SLE patients to create harmful products that alter immune regulation and trigger autoimmunity. The abundance of ROS correlates with disease activity, and the damaging cascade is controlled by antioxidant repair systems (Figure 1) [36]. The most common enzymatic antioxidants are peroxidase, SOD, CAT, heme oxygenase, GPx, GR, and thioredoxin reductase [37,38]. Non-enzymatic antioxidants include vitamins A, C and E, carotenoids, flavonoids, GSH, uric acid (UA), and various antioxidant minerals [39]. Glutathione (L-γ-glutamyl-L-cysteinylglycine) is required for many biochemical pathways, playing a significant role in maintaining and regulating the thiol-redox state of the cell. In the normal cell, >90% of the total GSH pool is in the reduced form, and <10% is in the oxidized form (GSSG) [40]. The GSH:GSSG ratio is a valuable tool to define OS because it correlates with disease activity in SLE patients [41]. 

### 4.1. SOD in SLE

The SOD is an active protease containing metallic elements and widely exists in animals and plants [21]. SOD is necessary for its ability to clean oxygen FRs, where its levels are a sensitive index of antioxidant performance [42]. SOD plays a vital role in the genesis of SLE by maintaining the balance of the body’s oxidation–antioxidation system [43]. Several SOD isoforms (SOD1, SOD2, and SOD3) have been found in human cells [44]. SOD1 is a metallo-detoxifying enzyme and a scavenger of the radical anion O_2_•− that exists primarily in the cytoplasmic, peroxisomal, and mitochondrial membrane space. SOD1 accounts for approximately 90% of SOD activity in eukaryotic cells [45]. The O_2_•− anion is converted to H_2_O_2_ by the action of copper/zinc SOD (Cu/ZnSOD) in the cytosol and manganese SOD (MnSOD) in the mitochondria. H_2_O_2_ is reduced to H_2_O by CAT- or GSH-supported GPx-1 in the cytosol and GPx-4 in the mitochondria.

### 4.2. GPx in SLE

GSH is the main non-enzymatic antioxidant and redox modulator of human cells. Through GPx, GSH can be oxidized to glutathione disulfide (GSSG) to reduce H_2_O_2_ to H_2_O. GSSG is reduced to GSH to maintain a sufficient antioxidant level [46]. The increase in OS reflects the lower enzymatic activity of CAT in SLE, favoring the accumulation of harmful H_2_O_2_. On the other hand, the oxidant products are considered potential neo-antigens that could be involved in the pathogenesis of SLE.

### 4.3. CAT in SLE

CAT is an important endogenous antioxidant enzyme characterized by detoxifying H_2_O_2_ to O_2_ and H_2_O, limiting the harmful effects of ROS [47]. CAT is considered an essential regulator of OS when chronic exposure to ROS may contribute to the development of SLE [48]. A decrease in CAT activity affects the oxidant–antioxidant balance, promoting the premature appearance of atherogenesis with severe vascular effects in SLE patients [49].

There are other organic metabolites with antioxidant capacity, such as uric acid (UA). UA results from the breakdown of ingested and endogenously synthesized purines and is excreted by the kidneys and the intestinal tract without further metabolism [50]. UA also functions as an antioxidant, protecting cells from the adverse effects of ROS [51]. The UA molecule is involved in a complex reaction and has protective functions under OS conditions [52].

## 5. Mitochondrial Function in SLE

Mitochondria are the principal organelles responsible for ATP production by integrating the glycolysis process in the cytosol and the Krebs cycle, electron transport, and OxPhos in mitochondria. Although most of the respiratory enzyme complexes (approximately 90 polypeptides) are encoded by nuclear DNA (nDNA), 13 are encoded by mitochondrial DNA (mtDNA). Therefore, the mtDNA copy number and expression of mtDNA-encoded polypeptides may be crucial for energy delivery to cells. In general, mtDNA replication and transcription are controlled by the mitochondrial transcription factor A (Tfam), and further regulated by the nuclear respiratory factor (NRF)-1/NRF-2 [53].

In glycolysis, the enzymes hexokinase-II (HK-II), glucose 6-phosphate isomerase (GPI), and phosphofructokinase (PFK) regulate the rate-limiting step and, together with glyceraldehyde 3-phosphate dehydrogenase (GAPDH), convert glucose into pyruvate, which is metabolized to Acetyl-CoA to enter the Krebs cycle. Under some extreme conditions (inadequate oxygen supply or impaired mitochondrial function), pyruvate is reduced to lactate with the help of lactate dehydrogenase and hypoxia-inducible factor 1 alpha (HIF-1α) [54].

Mitochondrial dysfunction and OS are typical of T cell-mediated autoimmune disorders such as SLE, as a failure to maintain self-tolerance leads to autoreactive T cells that recognize self-antigens [55]. Autoreactive CD4 T cell effector subsets require mitochondrial-derived ROS and increased glycolytic metabolism to drive T cell fate and function. Thus, eliminating them or increasing self-tolerance through functional immunosuppressive CD4 regulatory T cells (Tregs) are therapeutic alternatives for autoimmune diseases [56]. Additionally, decreasing the glycolytic activity or increasing the antioxidant activity of GSH and SOD to inhibit cytotoxic CD8 T cell responses could be advantageous. Current treatment options for T cell-mediated autoimmune diseases such as type 1 diabetes mellitus, multiple sclerosis, rheumatoid arthritis, and SLE include global immunosuppression, antibodies to deplete immune cells and anti-cytokine therapy, and while they effectively decrease autoreactive T cells, they also compromise other immune responses, increasing susceptibility to other diseases and complications [55,56,57].

## 6. Mitophagy in SLE

Mitochondria form a sophisticated and dynamic network for energy production, primarily through OxPhos in connection with glycolysis and fatty acid oxidation. During OxPhos, electrons are transported in the inner chain of the mitochondria to generate an H+ gradient, which is crucial for the final step of ATP generation. Electron leakage or damaged transport chains can lead to ROS formation. Some ROS are not neutralized and act on cell homeostasis, with low ROS levels contributing to cell proliferation and survival. However, at higher levels, their actions as protein/lipid oxidants and inducers of DNA damage contribute to tumorigenesis and apoptosis. The accumulation of faulty mitochondria directly affects energy production and cellular homeostasis, and cells may attempt to repair them through fusion/fission mechanisms or degrade them through mitophagy [58].

The analysis of autophagy in the peripheral B cells of patients with SLE was recently reported, demonstrating maximal activation in naïve B cells. In SLE, naïve B cells encounter a tolerance checkpoint after the faulty immune phenotyping of B cells upon exiting the bone marrow [59]. Thus, autophagy could represent a potential therapeutic target in SLE because it may be a clinically relevant mechanism of action of the commonly used immunomodulatory antimalarial hydroxychloroquine (HCQ). HCQ has been used clinically to treat SLE, although its exact mechanism remains elusive. Some studies have shown that myeloid-derived suppressor cells (MDSCs) play a vital role in the regulation of SLE. HCQ apparently promotes MDSC apoptosis by upregulating the expression level of CD81 in MDSCs. By this mechanism, HCQ appears to relieve lupus symptoms (Figure 1) [60,61].

## 7. Toll-like Receptors in SLE

B lymphocytes are central in autoimmune diseases because specific autoantibody patterns often define them and exhibit a loss of tolerance. SLE is a prototypical disease associated with B cell hyperactivity. In SLE patients, loss of B cell tolerance to self-antigens is intrinsically controlled in the cells by toll-like receptors (TLRs).

The TLRs are a family of receptors crucial for the induction of innate and adaptive immune responses. Nucleic acid ligands activate a subset of TLRs on endosomes. TLR7 and TLR9 recognize single-stranded RNA fragments and DNA sequences containing unmethylated cytokine–phosphate–guanosine motifs, respectively (Figure 1) [62]. TLR signaling promotes three key activities that on B cells can contribute to autoimmune diseases: (a) antibody production, (b) antigen presentation to T cells, and (c) cytokine production. Genetic association studies implicate TLR signaling in SLE [63]. A TLR7 variant capable of producing greater activation in childhood-onset SLE has even been described [64].

TLR7 stimulates the extrafollicular B cell response and the germinal center reaction implicated in autoantibody production and disease pathogenesis, whereas TLR9 is required to produce autoantibodies that recognize double-stranded DNA-associated antigens that are abundant in SLE and are the hallmark of the disease. SLE patients display phenotypes consistent with increased TLR7 signaling associated with elevated CD27-IgD double-negative B cells and the CXCR5-CD11c+ subset (DN2 B cells or age-associated B cells) [65]. Surprisingly, TLR9 appears to protect against SLE partly by its ability to limit the stimulatory activity of TLR7 [66,67]. Antagonism between TLR7 and TLR9 can occur within a single B cell if it expresses a B cell antigen receptor (BCR) that recognizes self-antigens comprising TLR7 and TLR9 agonists [68]. However, it was recently reported that TLR9 restricts the differentiation of B cells instructed by TLR7 in vitro [69]. Though the roles of TLR7 and TLR9 in B cell effector function in patients with SLE remain to be fully elucidated, their unique signaling characteristics in B cells could have beneficial therapeutic effects.

TLR7 expression is higher in women than men because of its location on the X chromosome. One X chromosome is usually inactive in women, but some genes on the X chromosome, including TLR7, always seem to escape inactivation. As a result, TLR7 is expressed bi-allelically in plasmacytoid dendritic cells (pDCs), monocytes, and B cells. Thus, TLR7 is expressed at higher levels in women’s cells than men’s. B cells from women exposed to TLR7 agonists in vitro differentiate more efficiently into CD27hi plasmablasts than B cells from men. This gender difference is not observed with the addition of TLR9 (gene encoding chromosome 3) agonists [70], which is consistent with the higher prevalence of SLE in women than in men [71]. The number of X chromosomes affects susceptibility to SLE. The presence of two X chromosomes in men with Klinefelter syndrome is associated with a greater predisposition to present SLE than in men with only one X chromosome [72]. Similarly, women with only one X chromosome (those with Turner syndrome) are less prone to SLE than women with two X chromosomes [48]. The reduction in the activity of TLR7 could lessen SLE development. TLR7 expression is also modulated by metabolic parameters (a high-fat diet), which exacerbates SLE [73].

It would be possible and desirable to reduce the symptoms of SLE by modulating the function of TLR7 [74]. However, circumstantial evidence supports enhanced TLR7 signaling as a mechanism of human systemic autoimmune disease [75]. TLR7 is a sensor for viral RNA and binds guanosine [76,77,78]. Viral nucleic acid sensors initiate vertebrate antiviral defense. Infections with RNA viruses, such as coronaviruses, influenza viruses, and Ebola viruses, activate extracellular or endosomal RNA sensors. TLR3, TLR7, and TLR8 sensors are specific for different RNA subtypes that constitute the viral genome or appear during viral replication or gene expression, and activate different cellular responses, such as necroptotic cell death, interferon signaling, and inflammation [79,80,81]. Mouse TLR7 and human TLRs 7 and 8 detect imidazoquinolines guanosine-based drugs that induce an antiviral response in vivo. TLRs 7 and 8 are close phylogenetic relatives that arose from an X-linked duplication event. However, the microbial ligands for TLRs 7 and 8 remain subject to considerable puzzlement [78].

## 8. Lupus Nephritis

One of SLE’s most severe organic manifestations is lupus nephritis (LN), a form of glomerulonephritis. Most patients with the disease develop LN within five years of diagnosis, and in many cases, LN is the initial diagnostic manifestation of the disease [82]. LN is one of the leading causes of morbidity and mortality in most patients at some point in their disease, and is characterized primarily by long-lasting, relentless proteinuria. Therefore, proteinuria is a severe and frequent SLE complication and a poor prognostic sign [83].

**Figure 3:**
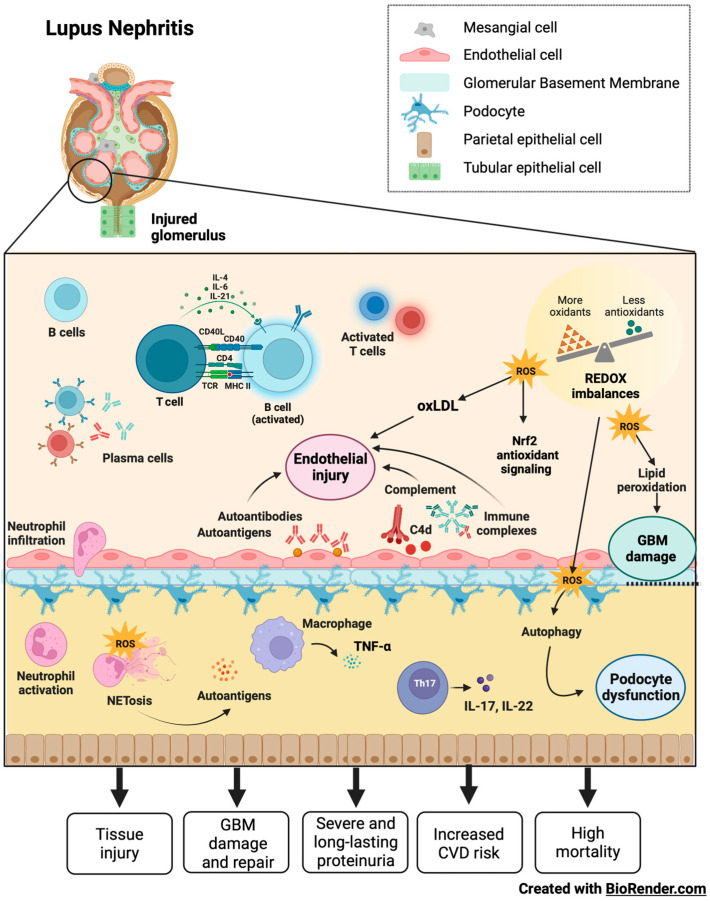
LN and its damage mechanism. Glomerular damage in LN features endothelial injury, glomerular membrane damage, and podocytes dysfunction mediated by autoantibodies, immune complexes, complement system activation, inflammation, autophagy, NETs, and redox imbalances. In patients with LN, a robust self-renewal and repair system of the glomerular filtration membrane results in instability regarding the degrees of renal injury and proteinuria. In early LN, it is possible to repair pathological damage to the filtration membrane caused by complement proteins and cytokines through autoregulation. However, tissue damage cannot be repaired as the disease progresses, leading to severe proteinuria, increased risk of cardiovascular disease, and high mortality.

LN patients have varying degrees of kidney injury and proteinuria levels are unstable, presumably because of robust glomerular filtration membrane self-renewal and repair systems (Figure 3) [84]. However, the injury cannot be fully repaired as the disease progresses, leading to severe proteinuria. It is urgent to explore the self-repair mechanism of the glomerular filtration membrane in early-stage LN and its failure mechanism in late-stage LN [85].

Podocyte-induced proteinuria is associated with pore membrane protein expression and autophagy in LN [86]. Podocytes are highly specific innate glomerular cells and play a role in the glomerular basement membrane filtration barrier, pore size, and energy charge, so searching for therapeutic targets to prevent podocyte damage is essential from a clinical perspective in treating LN [87].

Glomerular immune complex deposition and the activation of the complement system are involved in the pathogenesis of LN. Additionally; evidence suggests that OS significantly contributes to renal failure over time in SLE [88]. 

### 8.1. Oxidative Stress in LN

**Figure 4:**
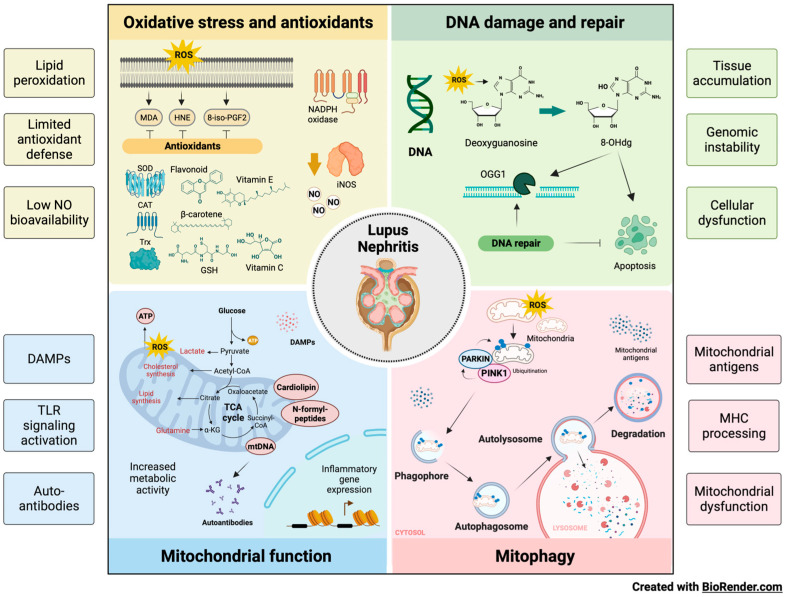
Influence of OS, DNA damage, antioxidants, mitochondrial function and mitophagy on the development of LN. Oxidation leads to organic damage characteristic of SLE, especially in LN. Increases in LPO due to OS, limited antioxidant defense, and low NO availability contribute to the injury observed in LN. The balance between oxidation and DNA repair is altered in patients with LN, accumulating the marker of oxidative DNA damage (8-OHdG), genomic instability, and cellular dysfunction. Additionally, metabolic abnormalities influence the nature and state of activation of the kidney’s immune cell infiltrate, highlighting the importance of mitochondrial function in developing diseases such as LN through Danger-Associated Molecular Patterns (DAMPs), TLR activation, and the production of autoantibodies. Finally, aberrant or defective mitophagy is central to the pathology of LN as it facilitates the release of mitochondrial antigens, promotes their processing by major histocompatibility complex (MHC) molecules, and contributes to mitochondrial dysfunction.

Oxidation is key in developing the organic damage characteristic of SLE, especially in LN (Figure 4) [89]. Patients with LN in the active phase have an unbalanced redox state that causes LPO of the glomerular basement membrane, altering its integrity and affecting tubular function. LPOs generate a variety of metabolites, the best-known being saturated aldehydes. In LN, high levels of OS were previously detected in patients with active SLE, suggesting the link between LPO and disease activity. Increased activities of MDA, HNE, MDA protein adduct, HNE protein adduct, SOD, inducible nitric oxide synthase (iNOS), anti-MDA, and anti-HNE antibodies correlate with the SLE Disease Activity Index (SLEDAI) in patients during follow-up [90].

LPO’s destructive effect is limited by the defense mechanisms of the human organism, such as the metabolization of LPO by oxide reductase and antioxidant enzymes, as well as vitamins and minerals [91]. F2-isoprostanes (8-iso-PGF2) levels are a result of LPO and are correlated with disease activity in subjects with LN [92].

The increase in protein carbonyls may result from the presence of multiple oxidants. Carbonyl formation indicates that total protein oxidation levels are high in patients with SLE and LN, thus suggesting the altered balance between oxidation and repair in these patients [90,93].

NO is a ubiquitous intra- and extracellular messenger molecule synthesized from L-arginine by a group of enzymes called NOS that is involved in various signaling pathways, including inflammation, immunity, ion channel modulation, gene transcription, and vascular tone [20,94]. There are three isoforms of NOS, often described based on their tissue expression: neuronal NOS (nNOS or NOS1), inducible NOS (iNOS or NOS2), and endothelial NOS (eNOS or NOS3) [95]. The kidney expresses all three isoforms, which are known to affect natriuretic and diuresis and contribute to blood pressure control [96]. Various renal cells can release NO, including endothelial and mesangial cells, the macula densa, and podocytes [97]. Since NO plays a complex role in ultrafiltration, vasodilation, and glomerular inflammation, changes in NO bioavailability lead to podocytes damage, proteinuria, and the rapid development of chronic kidney disease (CKD) under pathophysiological conditions such as hypertension or diabetes. Restoring NO levels may be beneficial for glomerular function. At the same time, the compromised activity of NOS enzymes can lead to the formation of ONOO−.

Changes in the distribution of NO sources due to the altered expression of NOS subunits or shifts in the activity of NADPH oxidases can link or promote the development of pathologies. However, the mechanisms describing NO’s production and release in glomerular cells, the interaction of NO and other ROS in podocytes, and how crosstalk between NO and calcium regulates glomerular cell function are still not fully described [98]. iNOS is upregulated in mouse models of LN and over-expressed in the glomerulus in proliferative human LN [99]. The administration of iNOS competitive inhibitors reduced reactive OS markers in two mouse models of LN, strongly suggesting that most systemic OS in these models results from iNOS activity. The relative swiftness of ROS reduction with iNOS inhibitors points to a direct effect on the iNOS enzyme, rather than the known impacts of more extended treatment with iNOS inhibitors on cell proliferation and glomerular infiltration in murine models [100]. It was previously reported that brief therapy with iNOS inhibitors does not significantly affect renal pathology, and the deletion of the iNOS gene in the absence of infection reduces systemic OS levels [101].

OS can inflict renal damage by promoting tumor necrosis factor-alpha (TNF-α) production by infiltrating macrophages, excessive Th17 cell differentiation, secretion of the inflammatory cytokine IL-17, and activation of neutrophils. By blocking TNF-α, it has been possible to alleviate OS and renal damage in NZB/NZW mice with interferon-alpha (IFNα)-induced LN [102]. 

Because kidney injury acts as a risk factor for CVD, OS is the basis of the pathogenesis of CVD in SLE. It is possible to observe an increased risk of atherosclerosis in patients with SLE compared to healthy individuals, and the disruption of lipid and lipoprotein metabolism induced by OS is of critical importance [103]. MDA-modified oxidized low-density lipoprotein (OxLDL), autoantibodies against endothelial cells, and phospholipids may also directly contribute to endothelial damage in early lupus or through the activation of the type I IFN pathway [104]. Monocytes phagocytosed OxLDL to differentiate into foam cells and release inflammatory substances, predisposing the subject to atherosclerosis. NETs are central components of neutrophils involved in OS with the ability to damage endothelial cells, activate macrophages release myeloperoxidase (NADPH oxidase capable of oxidizing HDL), and decrease levels of the antioxidant HDL [105]. 

However, OS is essential for forming anti-β2GPI antibodies and thrombotic events in patients with SLE. Moreover, the OxLDL/β2GPI/anti-2GPI complex induces the differentiation of macrophages in foam cells, which promotes the development of atherosclerosis, possibly through the mechanism by which HNE oxidizes the β2GPI antigen, which it enhances immunogenicity in response to increased anti-β2GPI antibodies and the aPL antibody cross-reacts with OxLDL, facilitating its entry into macrophages and promoting the progression of atherosclerosis [106]. Most atherosclerosis risk factors increase OS and accelerate disease progression. Arterial hypertension is associated with OS, and its presence in SLE implies a dysregulated ratio of Th1/Th2 cytokines, the increased production of IL-17, and insulin resistance. By augmenting the susceptibility of the tiny renal inlet arteries to angiotensin II and the expression of sodium chloride co-transporters, OS may enhance the reabsorption of water and sodium in the renal tubules, precipitating the development of hypertension [107,108].

Nuclear factor E2-related factor 2 (Nrf2) is a vital regulator of the antioxidant response in LN. Nrf2 has an antioxidant role that promotes cell protection and the prevention of tissue damage. It was recently revealed that the expression of Nrf2 and downstream molecules, heme oxygenase-1 (HO-1) and GPx, are downregulated in mouse models of diabetic nephropathy and IgA nephropathy [109]. 

### 8.2. Oxidative Damage to DNA in LN

Since the enzyme OGG1 is responsible for 8-OH-dG’s clearance, its deficiency results in high levels of DNA lesions by 8-OH-dG in DNA. OGG1 overexpression improves mitochondrial function and cell survival under OS conditions in vitro, highlighting its protective role in inflammatory diseases. Furthermore, the OGG1 polymorphism could offer susceptibility to LN and modulate the serum level of 8-OH-dG in patients with SLE [110]. 

### 8.3. Antioxidants in LN

Antioxidants protect against SLE development by opposing OS. Surface thiols and GSH participate in the redox quenching of cells, protecting against OS. In LN, chronic inflammation is associated with OS and decreased levels of the cellular antioxidant sulfhydryl [111]. The imbalance of the oxidative state, represented by an increase in plasma MDA and a reduction in GSH, is a possible cause of LN activity in SLE (Figure 4). However, treating LN with antioxidants is rarely documented [112,113]. 

N-acetyl cysteine (NAC) is a reliable antioxidant often applied for clinical treatment. In two recent cases of LN treated with antioxidants, the authors reported their beneficial effect in modulating oxidative status, though the underlying mechanisms require further investigation [114]. Studies have indicated that antioxidants can reduce oxygen consumption in mitochondria and block mTOR. In LN, antioxidants increase GSH and lower F2-8-isoprostane levels [115]. 

### 8.4. Mitochondrial Function in LN

The mitochondrion is an intracellular organelle that regulates numerous cellular functions, among which the best known are ATP production and programmed cell death [116]. Mitochondria are derived from the endosymbiosis of an α-proteobacterium and a precursor of the eukaryotic cell, giving the organelles many bacterial characteristics [117]. Unlike other organelles, mitochondria form by binary fission and cannot be produced directly by the cell. 

Mitochondria consist of an intermembrane space and a mitochondrial matrix separated by the outer mitochondrial membrane (OMM) and the inner mitochondrial membrane (IMM). Mitochondria are assembled through the interaction between the nuclear and mitochondrial genomes. Mammalian mitochondrial DNA (mtDNA) encodes 37 genes, 13 of which encode polypeptide components of the OxPhos machinery, and the 22 tRNAs and two ribosomes RNA (rRNAs) required for gene transcription and translation within the organelle [118]. Approximately 99% of mitochondrial proteins are encoded by nuclear genes, translated on cytoplasmic ribosomes, and imported into mitochondria by the translocase OMM and translocase IMM complexes [119].

Mitochondria are involved in the synthesis of fatty acids, the production of amino acids, the synthesis of heme, and the biogenesis of iron and sulfur groups [120]. Mitochondria communicate with the ER through the mitochondria-associated ER membrane (MAM) to regulate Ca^2+^ homeostasis, lipids, and apoptosis [121]. Mitochondria are an important site of ROS (mtROS) production. Under normal conditions, mtROS are rapidly cleared by the enzyme SOD2 in the mitochondrial matrix and SOD1 in the mitochondrial intermembrane space, as well as by other antioxidant enzymes, GPX, GSH, and glutathione disulfide [122]. 

Physiological levels of mtROS are essential for various signaling functions by maintaining the functional state of mitochondria, while excess mtROS causes oxidative damage to proteins, lipids, and DNA, leading to ATP depletion and a further increase in the production of mtROS and activation of the inflammasome, which exacerbates cell damage and initiates programmed cell death [123]. Mitochondria contain numerous copies of a compact circular genome that encodes RNA molecules and proteins involved in mitochondrial OxPhos. The mtDNA activates the immune system present in the cytosol or the extracellular environment. Because mitochondria retain several features of their ancestral prokaryotic origin, releasing mitochondrial components into the extracellular milieu can activate the innate immune system [124].

Cardiolipin, N-formylated peptides, mtDNA, ATP, and ROS, are known damage molecular patterns (DAMPs) associated with mitochondria that activate cells through nuclear oligomerization domain-like receptors, TLR-like receptors (e.g., TLR9 for mtDNA) or formyl peptide receptors. The mitochondria are also the target of circulating autoantibodies in SLE. However, whether mtRNA is also recognized by autoantibodies in SLE is unknown [125]. Anti-mitochondrial autoantibodies recognize proteins, such as those involved in OxPhos, phospholipids, or unidentified epitopes present on the mitochondrial membrane. Despite the extensive literature on antibodies directed to cardiolipin (mitochondrial M1 antigen) in SLE, the repertoire of anti-mitochondrial autoantibodies and their antigenic targets must still be characterized [126].

Metabolic abnormalities influence the nature and state of activation of the kidney’s immune cell infiltrate, highlighting the importance of mitochondrial function in developing diseases such as LN [127]. An analysis of gene expression profiles in lupus-affected human and mouse kidneys revealed increases in gene sets characteristic of myeloid cells, accompanied by decreases in genes that control glucose and lipid metabolism [128]. Even metabolism-linked transcriptional alterations were found in LN patients with less severe glomerular damage, indicating that metabolic dysfunction is an early and common change in lupus-affected tissues that results from immunological processes and contributes to tissue damage. At the same time, the expression of tubular damage markers was negatively correlated with the TCA cycle in murine models of LN. Likewise, transcriptional studies show that defects in regulating fatty acid oxidation in renal tubular epithelial cells facilitate important intracellular damage mechanisms in LN such as lipid deposition, ATP depletion, cell death, and fibrosis [129].

It has been described that glycolysis regulates macrophage polarization and the association between the intra renal presence of macrophage markers, with increased pentose phosphate pathway activity linked to renal dysfunction and increased cytokines in patients affected by SLE [130]. Similarly, T cells can increase glycolysis in response to their activation, and this increase, while essential to carrying out their effector functions, can lead to autoimmunity [131,132,133]. Some studies suggest that the highest percentage of kidney-infiltrating cells correspond to T cells with an activated phenotype [134,135]. However, recent evidence demonstrates that in LN, CD4 and CD8 T cells from renal tissue are not functional effector cells, but have a reduced ability to proliferate and produce cytokines [136]. This hypo functional phenotype observed in preclinical models of LN has been linked to the presence of mitochondrial dysfunction and an exhausted transcriptional signature.

Interferon-gamma (IFNγ) produced by CD4 T cells and nicotinamide phosphoribosyl transferase (NAMPT), a rate-limiting enzyme in the NAD+ biosynthetic pathway, are crucial elements in the pathogenesis of LN [137]. In CD4 T cells from LN patients or MRL/lpr NAMPT mice, aerobic glycolysis and mitochondrial respiration are promoted through the production of NAD+. The NAMPT inhibition suppresses IFNγ production in CD4 T cells, thus decreasing inflammatory cell infiltration and renal damage. NAMPT can potentially normalize the metabolic competence and pathogenicity of CD4 T cells in LN. It has also been observed that glycolysis normalization and oxidative metabolism in CD4 T cells by treatment with metformin and 2-deoxy-D-glucose leads to disease improvement in murine models of lupus [138]. This evidence supports the development of targeted therapies to control mitochondrial metabolism in T cell subsets to treat systemic autoimmune diseases such as LN.

### 8.5. Mitophagy in LN

Mitophagy is the process by which dysfunctional or superfluous mitochondria are selectively removed by autophagy to control their quality and quantity [139]. Recent mitophagy studies reveal that mitochondrial priming is mediated by the phosphatase and tensin homolog (PTEN) induced kinase 1 (PINK1)/Parkin signaling pathway and mitophagy receptors [140]. Mitophagy is potently induced during OS and facilitates mitochondrial quality control to mediate metabolic adjustments to external challenges. At the same time, impaired mitophagy is responsible for mitochondrial dysfunction and the progressive accumulation of defective organelles, leading to cell death and tissue damage. The mild and transient OS induced by H_2_O_2_ at low concentrations for a short time leads to low levels of ROS capable of inducing mitophagy, suggesting that mitophagy functions as an early cytoprotective response that favors OS adaptation by eliminating damaged mitochondria [141].

Once cellular mitochondria are damaged by increased OS and apoptotic proteases exceed the range that mitophagy can eliminate, the programmed cell death pathway is activated [142]. The balance between mitophagy and apoptosis plays a critical role in determining cell fate under conditions of OS, hypoxia, DNA damage, and loss of growth factors. The kidney is an energetically demanding organ rich in mitochondria; even renal function depends to a large extent on mitophagy [143]. Mitochondrial antigens can be generated by degrading old or damaged mitochondria through mitophagy. Mitochondria-containing auto phagosomes travel through the endo-lysosomal system, leading to the degradation of their cargo and allowing the production of mitochondrial peptides that can be processed and expressed by the major histocompatibility complex (MHC). Both MHC-I and MHC-II have been implicated. 

However, a recent study revealed that mitochondrial antigen processing can also occur independently of mitophagy. Mitochondrial antigens are transported to endosomes by mitochondria-derived vesicles formed by a mechanism regulated by PINK1 and Parkin proteins [144]. There is an intense effort to discover new biomarkers that make it possible to discriminate patients with SLE specifically. Anti-mtDNA antibodies are positively associated with nephritis, whereas anti-mtRNA antibodies show a negative association. AmtDNA and AmtRNA can help predict the risk of kidney damage in patients with SLE (Figure 4) [145].

Emerging evidence suggests that aberrant or defective mitophagy is central to many renal diseases, including LN pathology [146]. Multiple signaling pathways are involved in the deterioration of mitophagy in SLE and LN, so multi-targeted therapies are required to induce remission and prevent flare-ups. Rapamycin prevents LN development in lupus-prone mice [147] and patients with SLE [148] by inhibiting mTOR and enhancing auto phagosome formation and auto lysosomal degradation [149]. Recent studies have demonstrated the protective effects of rapamycin on mitochondrial function in the context of SLE. Rapamycin increases Drp1 through mTOR inhibition in lupus-prone mice and decreases mitochondrial dysfunction by activating mitophagy. Competent mitophagy may have therapeutic effects in LN, and drugs that induce mitophagy, such as rapamycin and 3-PEHPC, deserve further exploration as therapeutic strategies to enhance the clearance of fragmented mitochondria that promote injury and speed recovery from SLE and LN outbreaks [150].

In LN, the renal tissue of lupus mice and the serum of lupus patients exhibit atypically high levels of autophagy and autophagy markers, like programmed cell death-1 (Beclin-1) [151,152]. Moreover, the peripheral blood mononuclear cells from SLE patients and macrophages derived from murine lupus also express Beclin 1, and the adoptive transfer of Beclin 1-knocked down macrophages decreases anti-dsDNA antibodies, proteinuria, and renal immune complex deposition through reduced cytokine production [153]. Thus, autophagy inhibition by targeting Beclin 1 could represent an alternative for treating SLE and LN by limiting the excessive activation of pro-inflammatory macrophages.

### 8.6. Innate Immunity in LN

The pathogenesis of LN involves a variety of mechanisms. The extra renal etiology of SLE is based on multiple combinations of genetic variants that compromise the mechanisms that generally ensure immune tolerance to nuclear autoantigens. The loss of immune tolerance becomes clinically detectable by the presence of antinuclear antibodies. The oxidant/antioxidant imbalance in SLE due to exposure to endogenous or exogenous toxic factors produced by alterations of the response/repair mechanisms of tissue damage induces aberrant activity of the innate and adaptive immune response, with high production of autoantibodies and multiple lesions of the target tissues and organs [154]. 

The nucleic acid component of the immune complexes activates intra renal inflammation by TLRs in intra renal macrophages and dendritic cells. Immunostimulatory nucleic acids activate glomerular endothelium, mesangial cells, and macrophages to produce large amounts of pro-inflammatory cytokines, IFN-α, and interferon-beta (IFN-β) [155]. Nucleic acids released from the network or apoptotic neutrophils activate innate and adaptive immunity via viral nucleic acid-specific TLRs [77,78,79]. Therefore, many clinical manifestations of SLE resemble a viral infection. Endogenous nuclear particles activate IFN-α signaling in the same way as viral particles during viral infection. Dendritic, helper T, B, and plasma cells contribute to aberrant polyclonal autoimmunity. The intra renal etiology of LN involves binding antibodies to multiple intra renal autoantigens rather than the deposition of circulating immune complexes. Tertiary lymphoid tissue formation and local antibody production add to intra-renal complement activation as renal immunopathology progresses [156]. The delay in eliminating dead cells leads to the degeneration of their components, compromising the elements that distinguish self-nucleic acids from viral nucleic acids [157]. However, nature evolved DNA and RNA methylation to inhibit RNA and DNA recognition by TLRs 3, 7, and 9 (endosomal viral nucleic acid recognition receptor set), which trigger antiviral immunity during viral infection [158].

**Figure 5:**
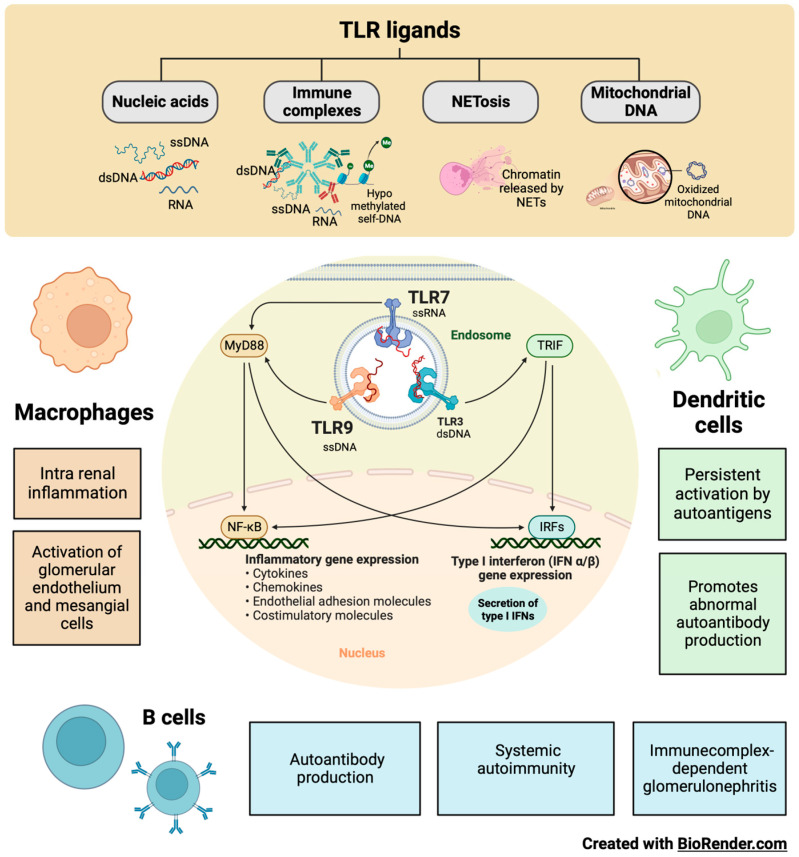
Participation of innate immunity and TLRs in the development of LN. Some TLRs, such as TLR3, TLR7, and TLR9, are in the endosomal compartment, where they detect endogenous ligands such as RNA, DNA, hypo-methylated self-DNA within immune complexes, chromatin released from NETs, oxidized mitochondrial nucleoids, and other chromatin formats. Dendritic and B cells can process antigens and present antigens to T cells. In LN, TLR7 and TLR9 allow the persistent activation of dendritic and B cells by autoantigens, thereby promoting autoantibody production, systemic autoimmunity, and glomerulonephritis mediated by immune complexes. In addition, the nucleic acid component of the immune complexes also activates intra renal inflammation via TLR7 and TLR9 in intra renal macrophages, resulting in the activation of glomerular endothelium and mesangial cells that are characteristic of LN.

Dendritic and B cells can process antigens and present antigens to T cells, and are able to substitute for one another for that purpose [159]. Dendritic cells have a limited lifespan, but their persistent activation by SLE autoantigens through TLR7 and TLR9 improves their survival and renders them resistant to glucocorticoid-induced killing [160]. The nucleic acid component of immune complexes also activates intra-renal inflammation via TLR in intra renal macrophages and dendritic cells (Figure 5) [161]. Immunostimulatory nucleic acids activate glomerular endothelium, mesangial cells, and macrophages to produce large amounts of pro-inflammatory cytokines IFN-α and IFN-β [162]. The functional importance of intra glomerular IFN signaling needs to be better understood. Still, it contributes to renal damage in LN, and would trigger the formation of tubule reticular structures or inclusions as an ultrastructural feature of IFN signaling [163]. The binding of TLRs and complement receptors activates renal cells to release pro-inflammatory cytokines and chemokines and induce the luminal expression of selectins and adhesion molecules within the microvasculature [164]. The concept of pseudo-antiviral immunity is based on the molecular mimicry of endogenous nucleic acids in the viral nucleic acid recognition receptors TLR7 and TLR9 [165]. TLR blockade and subsequent IFN signaling add to the established therapeutic targets in SLE [166]. 

### 8.7. Toll-like Receptors in LN

Current therapeutic approaches may fail due to their inability to direct distal innate immune responses to immune complex deposits adequately. Understanding the mechanisms that drive innate downstream responses is essential for developing new therapies for SLE and LN. A key mediator of innate immune responses is increased ROS production in response to inflammatory stimuli [167].

The local release of pro-inflammatory cytokines blocks TLRs with the capacity to improve the efficacy of treatment in autoimmunity without increasing systemic immunosuppression [168]. TLR9 is in the endosomal compartment, where it can detect unmethylated CpG motifs in endogenous DNA sequences or exogenous DNA from viruses or bacteria [169]. There are endogenous TLR9 ligands, such as hypo-methylated self-DNA, within immune complexes, NETs, oxidized mitochondrial nucleoids, and other chromatin formats [170]. Defects in lysosomal maturation support TLR9 activation [161]. Additionally, Type I IFNs promote receptor crosslinking responsiveness in B cells and make dendritic cells responsive to endogenous nucleic acids after upregulating TLR7 and TLR9 [171]. The blockade of endogenous TLR9 ligands with a TLR9 antagonist in MRL/lpr mice ameliorates systemic autoimmunity and immune complex glomerulonephritis [172]. Consistently, CpG and LPS application to anti-dsDNA transgenic mice exacerbates the disease [173]. Extrinsic and intrinsic ligands can bind TLRs on infiltrating monocytes, dendritic cells, and B cells to enhance cytokine secretion [174].

#### 8.7.1. TLR7 in LN

TLR7s were first described as innate pathogen recognition receptors that trigger appropriate antimicrobial immune responses upon exposure to pathogen-associated molecules. In parallel with ongoing studies on TLR biology, growing experimental evidence suggests that endogenous RNA-related self-antigens may also activate dendritic and B cells via TLR7. TLR7-mediated dendritic cell activation, autoantibody secretion, lymph proliferation, and autoimmune tissue injury are frequently observed in several murine models of SLE and LN (Figure 5) [175]. 

It was recently reported that polymorphisms in TLR7 are related to the development of lupus [176]. Murine models of lupus have provided genetic and experimental evidence to support a role for TLR7 activation in LN pathogenesis [177]. Male BXSB mice, which contain an extra copy of TLR7 on the Y chromosome, develop LN, whereas females are protected [178]. Transgenic mice that overexpress TLR7 also develop lupus [179]. Murine lupus models, such as the pristane-induced lupus model, depend on TLR7 signaling for lupus pathogenesis [180]. Repeated epicutaneous application of the TLR7 agonist to wild-type mice leads to lupus-like features, including mild LN. Despite these supporting data, the mechanisms by which TLR7 signaling leads to LN remain unclear [181]. 

TLR7 activation can result in the production of type I IFNs and the activation of nuclear factor kappa B (NFκB) in various cell populations, including dendritic cells, monocytes, macrophages, and B cells. Type I IFNs, including IFNα, can promote the development of lupus and are sufficient to accelerate nephritis in lupus-prone mice [176]. Several genetic and inducible models are protected from developing lupus when type I IFN signaling is knocked down [182].

A moderate increase in TLR7 is sufficient for developing nephritis, while the normalization of B cell TLR7 expression or temporary pDCs depletion slows the progression of LN. The conventional dendritic cell expression of TLR7 is essential for severe autoimmunity in SLE. A new expanding CD11b(+) conventional CD subpopulation dominates the renal infiltrating inflammatory milieu with localization in the glomeruli. The exposure of human myeloid dendritic cells to IFN-α or Flu increases TLR7 expression, suggesting that they may have a role in self-RNA recognition pathways in clinical disease [183]. 

TLR7 recognizes single-stranded RNA (ssRNA), which induces the downstream activation of signaling molecules, including Jnk and NFκB, through a myeloid differentiation primary response gene 88 (MyD88)-dependent cascade. This process is essential for the host’s defense against invading viruses. However, TLR7 hyperactivity may also drive the initiation and progression of autoimmunity [184]. TLR7 and the MyD88 signaling pathway are critical for initiating autoimmunity and developing autoreactivity because their genetic ablation prevents the development of anti-nuclear antibodies and immune pathology. This signaling pathway is required explicitly within B cells [185]. 

TLR7 has emerged as a significant regulator of autoantibody production in murine LN. Tonic interactions between TLRs and environmental agonists derived from commensal microbes and endogenous sources may also influence autoimmune diseases and inflammatory disorders affecting the kidney [186]. The contributions of TLRs and other innate immune receptors in regulating inflammation, immune responses, and kidney injury remain to be elucidated. 

#### 8.7.2. TLR9 in LN

In recent studies of selective TLR9 deletion or over-expression, TLR9 deficiency in B cells was sufficient to exacerbate LN while quenching anti-nucleosome antibodies, whereas TLR9 deficiency in dendritic cells, pDCs, and neutrophils had no discernible effect on disease. B-cell-specific TLR9 deficiency appears to decouple the condition from autoantibody production, thus emphasizing the non-redundant role of TLR9 in B cells and its therapeutic potential [66]. TLR9 and TLR7 have paradoxical effects on the pathogenesis of SLE, especially since both receptors involved in downstream signaling pathways are believed to be nearly identical (Figure 5). 

Alternatively, and non-exclusively, TLR9 can regulate TLR7 in a cis manner within the same cell type by competing for shared rate-limiting downstream signaling components [187]. It was recently reported that, although TLR9 overexpression had a significant protective effect in two disease models, the scope of this protection may be limited by technical aspects related to the models used. TLR9 overexpression by both alleles did not occur in an average of 15.6% of B cells, so some B cells lacked the suppressive effect and might have dominantly promoted the disease by serving as antigen-presenting cells for autoreactive T cells. There is a precedent for this effect from escaped B cells when CD19-Cre was used to knock down MHCII in MRL/lpr mice conditionally [188]. Systemically administered TLR9 agonists have been used in clinical trials in the treatment of cancer, and were generally well tolerated [189]. TLR9 agonists could be designed only to activate B cells or even DNA-specific B cells. Therefore, understanding how cell populations regulate SLE could allow for a more targeted therapeutic design aimed at LN.

## 9. Management of SLE and LN

The early and accurate diagnosis of LN and early initiation of therapy is of vital importance to improve outcomes in patients with SLE [190]. The primary goal of treatment for this immunologic disease includes long-term patient survival, the prevention of target organ damage recurrences, and the optimization of health-related quality of life. Treatment generally consists of an initial period of high-intensity immunosuppressive therapy to control disease activity, followed by a more extended period of less-intensive therapy to consolidate the response and prevent relapses. Managing disease and treatment-related comorbidities, especially infections and atherosclerosis, is paramount. Conventional agents and new disease-modifying biologic agents, alone, in combination, or sequential, have improved rates of short- and long-term treatment goal achievement, including the minimization of glucocorticoid use [191]. 

**Figure 6:**
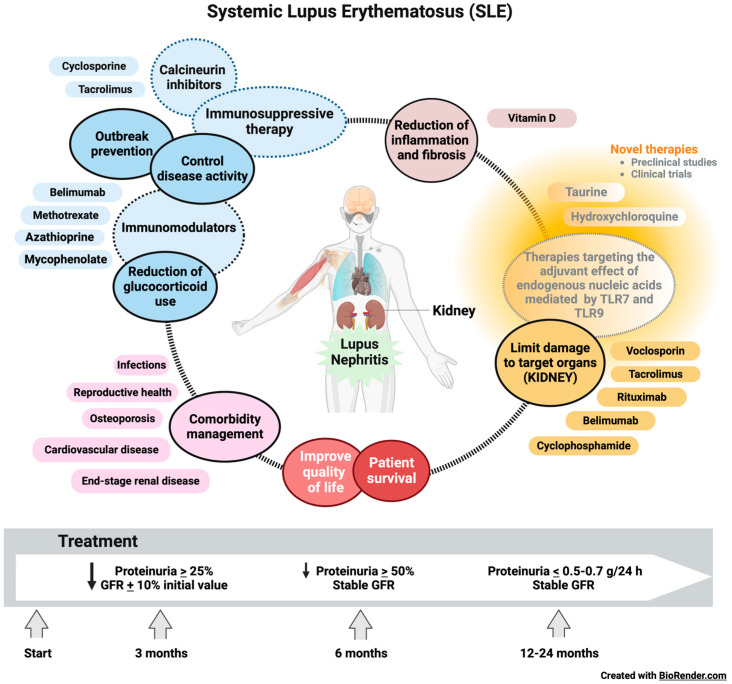
Therapeutic objectives and management of LN in SLE. The goal of therapy for LN was established with a reduction in proteinuria by ≥25% with a stable glomerular filtration rate (GFR, ±10% of baseline) in the first three months after the start of treatment, a reduction in ≥50% proteinuria at six months and a rate of proteinuria <0.5–0.7 g/24 h at 12–24 months (all with stable GFR). Immunosuppressive therapies, immunomodulatory drugs, and vitamin D are currently used to control disease activity, prevent relapses, reduce the use of glucocorticoids, and lessen inflammation and fibrosis. Voclosporin, tacrolimus, rituximab, belimumab, and cyclophosphamide limit damage to target organs, including the kidney, in patients with SLE. However, new therapies focused on reducing the effect of endogenous nucleic acids mediated by TLR7 and TLR9 present promising results in preclinical studies, suggesting that their translation to the clinic could significantly benefit the quality of life, survival, and management of comorbidities in patients with SLE and LN.

The goal of LN therapy was established with a reduction in proteinuria by ≥25% with a stable glomerular filtration rate (GFR, ±10% of baseline) in the first three months after the start of treatment, reduction in ≥50% proteinuria at six months and a rate of proteinuria of < 0.5–0.7 g/24 h at 12-24 months (all with stable GFR) (Figure 6) [192]. All SLE patients with LN should receive hydroxychloroquine at a dose not exceeding 5 mg/kg of actual body weight [193]. Antimalarial drugs such as hydroxychloroquine inhibit lysosomal acidification, thereby blocking the adjuvant effect of endogenous nucleic acids by TLR7 and TLR9 during the lysosomal processing of nuclear particles in the endolysosomal compartments of antigen-presenting cells [194].

During chronic maintenance treatment, glucocorticoids should be minimized to <7.5 mg/day (prednisone equivalent) and withdrawn when possible. The appropriate initiation of immunomodulatory agents (methotrexate, azathioprine, and mycophenolate mofetil (MMF)) may accelerate the tapering/discontinuation of glucocorticoids. In persistently active or exacerbating disease, belimumab should be considered as an add-on therapy for adult patients with active LN. Calcineurin inhibitors (voclosporin and tacrolimus) also show promising results for SLE with renal manifestations [195]. Even rituximab or cyclophosphamide (CY) may be considered in refractory organ-threatening disease [196]. It is a fact that the immunosuppressive therapy commonly used to treat SLE improves the LN condition and patient outcomes. However, kidney damage is not reversible [197]. Despite increasing knowledge regarding the pathogenesis of the disease and the availability of better treatment options, within ten years of the initial diagnosis, 5–20% of patients with LN develop end-stage renal disease (ESRD) with multiple comorbidities associated with the immuno suppressive therapy used to treat the underlying condition, including infections, osteoporosis, cardiovascular effects, and reproductive effects [10]. 

Our understanding of the pathophysiological manifestations of renal function in LN has improved substantially in recent decades. Even more specific TLR7 and TLR9 agents that effectively suppress LN in mouse models of SLE have been developed and are now in clinical trials [198]. In active proliferative LN, initial treatments (induction) with low-dose intravenous CY (500 mg × 6 biweekly doses) or MMF, 2–3 g/day, or mycophenolic acid at an equivalent dose, both combined with glucocorticoids (pulses of intravenous methylprednisolone, then oral prednisone 0.3–0.5 mg/kg/day), have been shown. The combinations of MMF with calcineurin inhibitors or high doses of CY are considered alternative regimens for patients with nephrotic range proteinuria and adverse prognostic factors, subsequent long-term maintenance treatment with MMF or azathioprine [199].

Minimizing patients’ exposure to glucocorticoids has received more attention after IV methylprednisolone pulses, the recommended starting dose being 0.3–0.5 mg/day prednisone equivalent. Prednisone should be gradually reduced to ≤7.5 mg/day at 3–6 months [200]. Due to its immunomodulatory and antifibrotic effects, vitamin D should be supplemented in all SLE patients with insufficiency or deficiency. The immunomodulatory properties of vitamin D are mediated by the vitamin D3 receptor (VDR) on multiple lineages of immune cells, including monocytes, dendritic cells, and activated T cells, as well as in the skin, vasculature, and other tissues. In vitro, vitamin D exerts an anti-inflammatory and anti-proliferative effect by promoting Th1 (TNF-α, IL-2, IFN-γ) to Th2 (IL-4, IL-5, IL-10, GATA3) polarization, as well as the change from Th17 (IL12, IL23, IL-6, 17) to Treg (IL-10, TGF-β, FoxP3, CTLA4) status. Additionally, vitamin D affects the development and function of NKT cells [201].

Taurine administration improved renal function, reversed cell death, suppressed OS, and adjusted the immune response of LN mice to a more balanced state. Taurine could be considered a novel strategy as a therapy in LN, which could overcome the disadvantages of traditional immunosuppression and hormone treatments with greater efficacy and fewer side effects [202]. Therefore, more studies are required on the mechanism of action of taurine, as there is an urgent need for therapies involving renal function recovery for patients with severe and terminal LN [199].

Luteolin has also been identified as a possible therapeutic option for preventing and treating LN due to its effect of suppressing the expression of HIF-1α in macrophages [203]. The bidirectional relationship between OS and the immune response could change the paradigm for diseases characterized by the perturbation of the immune system and the high production of autoantibodies [204].

## 10. Conclusions

SLE is a complex multifactorial autoimmune disease characterized by multiple and diverse cellular and molecular aberrations. The pathogenesis of SLE, like other autoimmune diseases and diabetes mellitus, is still far from being fully understood. The dysregulated immune response in SLE has been extensively studied, including innate and adaptive immunity. The B lymphocyte plays a central role in the production of autoantibodies, presentation of autoantigens, and activation of autoreactive T cells.

T lymphocytes participate by activating signaling pathways mediated by costimulatory and cytokines secreted by T cell subsets. When innate and adaptive immunity is activated, T cells also activate autoreactive B cells, which promote the deposition of immune complexes in tissues. Understanding SLE’s immune pathophysiology has led to new biological agents that specifically target abnormal immune processes that reduce unwanted adverse events associated with conventional broad-spectrum immunosuppressive therapies. Although our understanding of SLE remains incomplete and most new drugs are in clinical trials, they may lead to the development of safer and more effective therapies.

The precise characterization of the SLE phenotypes based on the molecular and clinical characteristics is crucial to designing a more personalized treatment, which can help redefine how LN is classified and will facilitate the identification of more precise predictors of the response to treatment. We hope that understanding the heterogeneity of auto-immunity in the behavior of SLE will soon lead to more effective and less toxic regimens that favor the clinical response of patients.

## Data Availability

No new data was created.
